# Development and pilot testing of HIV screening program integration within public/primary health centers providing antenatal care services in Maharashtra, India

**DOI:** 10.1186/1756-0500-7-177

**Published:** 2014-03-26

**Authors:** Suchitra V Bindoria, Ramesh Devkar, Indrani Gupta, Virupax Ranebennur, Niranjan Saggurti, Sowmya Ramesh, Dilip Deshmukh, Sanjeevsingh Gaikwad

**Affiliations:** 1 FHI 360, Bandra (East), Mumbai 400051, India; 2 Maharashtra State AIDS Control Society Wadala, Mumbai 400031, India; 3 Institute of Economic Growth, University Enclave, University of Delhi North Campus, Delhi 110007, India; 4 Population Council, 142, Golf Links, New Delhi, 110003, India

**Keywords:** Integration, Health programs, HIV screening, Antenatal care, PPTCT, Cost and cost-effectiveness

## Abstract

**Background:**

The objectives of this paper are: (1) to study the feasibility and relative benefits of integrating the prevention of parent-to-child transmission (PPTCT) component of the National AIDS Control Program with the maternal and child health component of the National Rural Health Mission (NRHM) by offering HIV screening at the primary healthcare level; and (2) to estimate the incremental cost-effectiveness ratio to understand whether the costs are commensurate with the benefits.

**Methods:**

The intervention included advocacy with political, administrative/health heads, and capacity building of health staff in Satara district, Maharashtra, India. The intervention also conducted biannual outreach activities at primary health centers (PHCs)/sub-centers (SCs); initiated facility-based integrated counseling and testing centers (FICTCs) at all round-the-clock PHCs; made the existing FICTCs functional and trained PHC nurses in HIV screening. All “functional” FICTCs were equipped to screen for HIV and trained staff provided counseling and conducted HIV testing as per the national protocol. Data were collected pre- and post- integration on the number of pregnant women screened for HIV, the number of functional FICTCs and intervention costs. Trend analyses on various outcome measures were conducted. Further, the incremental cost-effectiveness ratio per pregnant woman screened was calculated.

**Results:**

An additional 27% of HIV-infected women were detected during the intervention period as the annual HIV screening increased from pre- to post-intervention (55% to 79%, p < 0.001) among antenatal care (ANC) attendees under the NRHM. A greater increase in HIV screening was observed in PHCs/SCs. The proportions of functional FICTCs increased from 47% to 97% (p < 0.001). Additionally, 93% of HIV-infected pregnant women were linked to anti-retroviral therapy centers; 92% of mother-baby pairs received Nevirapine; and 89% of exposed babies were enrolled for early infant diagnosis. The incremental cost-effectiveness ratio was estimated at INR 44 (less than 1 US$) per pregnant woman tested.

**Conclusions:**

Integrating HIV screening with the broader Rural Health Mission is a promising opportunity to scale up the PPTCT program. However, advocacy, sensitization, capacity building and the judicious utilization of available resources are key to widening the reach of the PPTCT program in India and elsewhere.

## Background

Prevention of parent-to-child transmission(PPTCT) remains one of the key components of global HIV prevention effort [1]. HIV counseling and testing are a fundamental part of PPTCT programs and serve as an important entry point for the provision of HIV services [2,3]. However, despite several global initiatives and concerted national efforts over the last decade, majority of pregnant women are not aware of their HIV status [4]. The non-availability of on-site HIV testing facilities, especially at the primary health care level, results in missed opportunities for enrollment in the PPTCT program [5]. The success of the PPTCT program requires bridging gaps in coverage and providing effective preventive, diagnostic and therapeutic services in primary health care settings [6].

The integration of programs working in broadly similar areas is now seen as an important tool for achieving program efficiency. Establishing synergies and linking HIV programs with related health programs can be instrumental in effectively scaling up both programs [7-10].

The global health strategy for HIV and AIDS recommends strengthening health systems by adopting cost-effective, integrated service delivery models focusing on expanding access to quality HIV services [7]. Estimating the costs involved in any innovative model is essential as precedence must be given to cost-effective approaches leading to maximum HIV screening [4]. Specifically, such an approach requires strengthening the linkages between HIV and maternal and child health (MCH) programs at the primary care level, and task shifting to enhance the utilization of human resources. In developing countries like India, MCH activities are a vital part of health programs and PPTCT needs to be dovetailed with such programs for better outcomes.

In 2010, an estimated 35% of pregnant women received an HIV test in low and middle-income countries, up from 7% in 2005 [4]. In India, only 23% of pregnant women tested for HIV [4], while in Maharashtra, according to the program data 55% (1,196,254) [11] of pregnant women were screened for HIV in the year 2010–11, indicating a low screening volume.

The objectives of this paper are: (1) to study the feasibility of integrating the PPTCT component of the National AIDS Control Program (NACP) with the MCH component of the National Rural Health Mission (NRHM) by offering HIV screening at the primary health care level and assessing its benefits and impact in increasing HIV reactive diagnoses; and (2) to estimate the incremental cost-effective ratio to understand whether the costs are commensurate with the benefits.

## Methods

### Intervention setting

Until recently, the PPTCT program [12], a component of NACP [13] under the National AIDS Control Organization (NACO) [14], was being conducted in parallel with the MCH component of the NRHM program. The PPTCT program is rolled out through integrated testing and counseling centers (ICTCs) located at tertiary/secondary level government hospitals, facility-based ICTCs and public-private partnership facilities. The NRHM provides MCH services at all levels of health care; in particular, antenatal care (ANC) at village-level primary health centers (PHCs)/ sub centers (SCs) through village nurses assisted by a cadre of community health care workers called accredited social health activists (ASHAs) [15]. In 2010, the Ministry of Health and Family Welfare (MOHFW) and NACO called for the convergence of NACP with its flagship program, NRHM [16].

**Figure 1:**
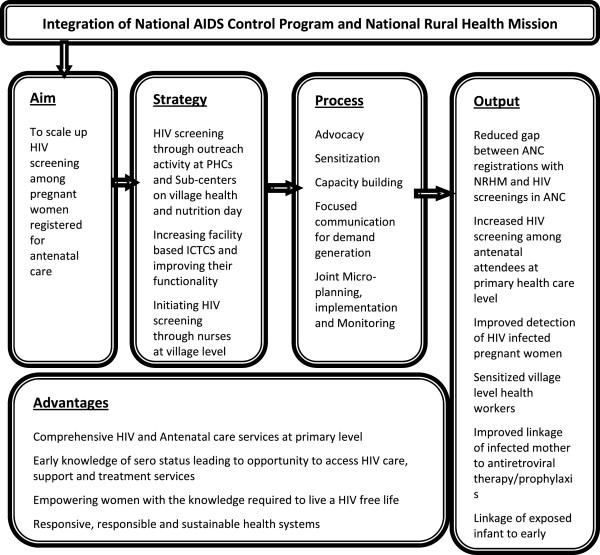
NACP -NRHM integration – the Satara pilot project.

The Maharashtra State AIDS Control Society (MSACS) [17], working with NACP across 33 districts in Maharashtra (one of the six high prevalence states in India), piloted a project to integrate HIV screening at NRHM primary health care level facilities in Satara district. This district, which is predominantly rural, has traditionally been a high HIV prevalence districts with an antenatal HIV prevalence of 1.63% in 2007 and 0.50% in 2008 [18]. The intervention included biannual outreach activities at PHCs/SCs, initiating facility-based ICTCs (FICTCs) at all round-the-clock PHCs (24X7 PHCs), making existing FICTCs functional and, subsequently, training PHC/SC nurses to screen for HIV at the village level. ”Functional” means that the ICTC is equipped to screen for HIV, and has trained staff to provide pre- and post-test counseling, carry out HIV testing as per the national protocol and routinely provide HIV screening (Figure 1).

The project was initiated with a situation assessment of HIV in the district, followed by advocacy and sensitization of district political, administrative and health heads. These district leaders mobilized and directed trained staff at PHCs to initiate FICTCs at their centers and to ensure their functionality. A cascade model was adopted for the training and sensitization of health and other staff. A joint plan for outreach activity at each PHC/SC was developed to coincide with the antenatal clinic day/village health and nutrition day. Focused IEC materials were used to create awareness and generate demand among eligible pregnant women (that is, all pregnant women who had not been tested for HIV at a government facility during their current pregnancy). The pilot project was formally launched by the district guardian minister and administrative head. The ASHAs mobilized eligible pregnant women to visit PHCs/SCs where they were provided ANC and voluntary HIV counseling and testing, after obtaining informed consent. Mobile ICTCs were used to facilitate HIV screening in hard-to-reach areas. Post-test counseling was carried out and results were shared on the same day. Early linkages with the nearest anti-retroviral therapy (ART) centers were facilitated through counselors and nurses. MSACS and NRHM officials regularly monitored the process.

To garner the benefits of the sensitized system and to ensure sustainability of the integration process, training on HIV screening using rapid testing (based on NACO protocol) was conducted for PHC/SC nurses at the village level following the second phase of outreach activities. The village nurses soon initiated HIV screening at the village level.

To summarize, the approach adopted to integrate HIV screening at the primary health level was through advocacy with district political/administrative/health heads, sensitization and capacity building of health staff, generating demand through focused communication, joint micro-planning to ensure better access to HIV counseling and testing, use of rapid HIV testisting at the village level and monitoring the program.

### Data

The reference period for the intervention was April 2011 to March 2012. The preceding year was considered as the benchmark or the pre-intervention period. Data on outreach activities were collected on a weekly basis and careful expenditure monitoring of all the items were undertaken during the pilot phase. Routine program data on HIV screening with health facility level break-up and cost data collected and compiled by the District AIDS Prevention Control Unit (DAPCU), Satara were used for analysis. The information gathered from the monitoring data used in the current study did not include individual identifiers.

The main outcome indicator to measure the feasibility and acceptability of integration was the pre- and post-intervention increase in HIV screening among ANC attendees. The benefits of integration were measured in terms of additional ANC women detected as HIV reactive, responsiveness of the district authorities, sustainability of the intervention and sensitization of health workers. Direct indicators were not available to measure all the benefits; hence, proxy indicators were used. The proportionate increase in HIV-infected pregnant women diagnosed was measured. The responsiveness of the district authorities was measured as the pre- and post-intervention difference in the following variables:

i. Number of functional facility-based ICTCs.

ii. Number of ANC attendees screened at PHCs/SCs.

iii. Number of ANC attendees screened for HIV by nurses at the village level.

The proportionate gap between ANC registration and HIV screening measured each month was assumed to reflect the sustainability of the intervention, while the gap in the period excluding outreach activity was used to measure the sensitization of NRHM health staff. Additionally, sensitization was indirectly measured as the proportion of ANC attendees screened for HIV by nurses at the village level.

The incremental costs were calculated, and the incremental cost-effectiveness was estimated based on the difference in the number of ANC women screened for HIV before and after the intervention. The major cost items are as follows:

•Communication materials– posters, banners and handouts.

•Training medical officers – training material and logistics.

•Travel–mobile vans deployed, counselors/technicians funded for travel, fuel, and maintenance of mobile vans.

•Documentation.

The project did not involve any additional costs for human resources, infrastructure and supplies like HIV testing kits as the resources available with MSACS/DAPCU and district NRHM were utilized. The travel costs of counselors/ laboratory technicians for outreach activities were covered by DAPCU under the routine travel budget. Thus, the only *additional* cost incurred was the temporary deployment of mobile vans, training of medical officers, printing of communication material and documentation.

### Statistical analyses

Simple descriptive statistics such as proportions, ratios and means were used to present the results. Differences in proportions were tested using Z-tests and Fisher’s exact test, and the differences in mean values were tested using t-tests. Trend analyses were conducted to measure the proportionate gap between women registered for antenatal care and those screened for HIV, functional FICTCs, ANC attendees screened at PHCs/SCs and village level HIV screening of ANC attendees by NRHM nurses. Cost-effectiveness was analyzed as the incremental cost-effectiveness ratio per pregnant woman screened.

## Results

A total of 42,120 ANC attendees were screened for HIV at the end of the intervention period, of which 11,223 (27%) were tested through the biannual outreach activity of the program (not shown in tabular format). Annual HIV screening of ANC attendees increased from 55%pre-intervention to 79% post-intervention (p < 0.001) (Table 1). Average HIV screening increased from 2,419/month pre-intervention to 3,510/month post-intervention (p < 0.001). Of those who tested HIV-positive, 70 were detected in the pre-intervention period while 89 were detected at the end of the intervention period: that is, a 27% increase in the detection of HIV-infected women post-intervention.

**Table 1:** HIV screening among pregnant women registered with NRHM, Satara district, Maharashtra, India

**Indicators**	**Pre-intervention**	**Post-intervention**	**P value**
**Total number of ANC registrations under NRHM**	**52411**	**53239**	
% women screened for HIV under NRHM^a, c^	55. 4	79.0	<0.001
**Number of functional PHCs in the district**	**36**	**36**	
% functional PHCs with NRHM supported ICTC facilities^a, d^	47.2	97.2	<0.001
**HIV screening by type of ICTC**^b^			
**Number of women screened for HIV**	**29022**	**42120**	
% screened at NACO-supported ICTCs (district/rural hospitals)^e^	86.3	75.0	
% screened at NRHM-FICTCs (PHCs/SCs/village)^e^	13.7	23.1	<0.001
% screened at public-private partnership ICTC clinics^e^	0.0	1.9	
**HIV screening at NRHM FICTCs**^b^			
**Number of women screened under NRHM -supported ICTCs**	**3982**	**9716**	
% tested at PHC by laboratory technician^f^	100	74.7	<0.001
% tested at SC/village by nurse^f^	0	25.3	

ANC: antenatal care; SC: sub-center; ICTC: integrated counseling and testing center; PHC: primary health center; FICTCs: facility-based integrated counseling and testing center.

^a^Z-test.

^b^Fisher’s exact test.

^c^Among pregnant women registered for ANC at NRHM health facilities.

^d^Among the number of 24X7 PHCs.

^e^Among total number of pregnant women screened for HIV.

^**f**^Among pregnant women screened for HIV at NRHM facility-based ICTC (PHCs/SCs/village).

As indicated in Table 1, the proportion of functional FICTCs at 24X7 PHCs rose from 47% pre-intervention to 97% post-intervention (p < 0.001). HIV screening at NRHM-PHCs/SCs increased from 14% pre-intervention to 23% post-intervention (p < 0.001). Village-level HIV screening initiated by nurses during the intervention period accounted for 6% of the total HIV screening and 25% of screening at the NRHM primary health care level during the post-intervention period (p < 0.001).

**Figure 2:**
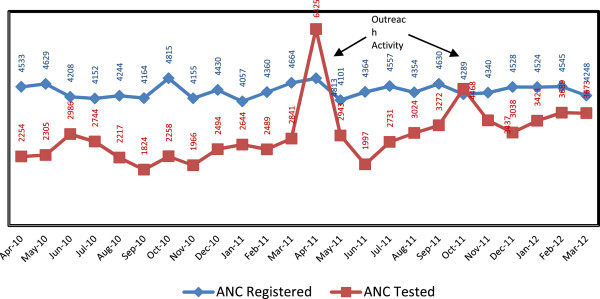
Decreasing gap between ANC registration and HIV screening among antenatal care attendees, Satara district.

The proportionate gap between ANC attendees registered and screened for HIV through the NRHM progressively decreased from 39% in April 2011 to 14% in March 2012.This can be seen in Figure 2 which shows the monthly movement of the variable over the two years, with the gap narrowing between ANC registration and HIV screening. The main spike in April 2011 is due to the outreach activity of the program in phase 1, And the shorter spike in October 2011 is due to outreach activity in phase 2.

The difference in HIV screening numbers in ANC attendees before and after the intervention was 13,098 (29,022 in 2010–11 and 42,120 in 2011–12) and the total incremental cost required to implement the project was INR 576,540 (approximately US$ 10676, 1 US$ = 54 INR), which included training costs (INR 19,800), cost of printing and distribution of communication materials (INR 491,320), travel costs (INR 61,560) for deployed mobile vans inclusive of fuel, vehicle maintenance, and travel allowance for accompanying drivers/counselors/technicians, and documentation costs (INR 3,600). Table 2 indicates an incremental cost-effectiveness ratio of INR 44 (less than US$1) per pregnant woman tested.

**Table 2:** Incremental cost-effectiveness ratio, Satara district, Maharashtra, India

	Costs^* ^**(INR)**	**Outcome: no. of women screened**	**Incremental cost -effectiveness ratio (INR)**
**Pre-intervention**	-	29022	576540/13098 = 44.01
**Post-intervention**	576540	42120	

INR – Indian rupees.

^*^Cost items included the cost for printing of communication materials, training of medical officers, travel to the field, and documentation.

In addition, as a result of integration where the primary health system was equipped with required resources, 93% of HIV-infected women detected post-intervention were linked to ART centers; 92% of mother-baby pairs received Nevirapine as per the national protocol while 89% of exposed babies were enrolled in a nearby infant diagnosis program (not presented in tabular format).

## Discussion

The study results suggest that the integration of HIV screening at the primary health care level as part of a comprehensive MCH program is feasible, beneficial, enhances the uptake of HIV testing, and facilitates wide geographic coverage. In addition, the incremental cost-effectiveness ratio post-intervention of INR 44 (less than US$1) per pregnant woman tested demonstrates that the integration of HIV screening at the primary health care level is a low-cost approach and brings a large number of pregnant women under HIV prevention services. In the absence of alternative cost data for achieving the same target of expanding HIV screening, the incremental cost-effectiveness ratio can be used as a proxy indicator to understand the financial implications of scaling up HIV screening using an integrated approach.

Integrating HIV screening with regular village health and nutrition days significantly scaled up HIV screening among pregnant women with the judicious utilization of the available resources within the two vertical health systems. In addition, this approach built the capacity of village-level health care workers, such as PHC/SC nurses and ASHAs, on HIV prevention and PPTCT issues. Sensitization of village-level nurses and health workers along with focused communication activities to generate a demand in the community led to an increase in HIV screening.

Advocacy and sensitization of district political, administrative and health heads have led to a more responsive, responsible and sustainable health system. These activities resulted in increasing the number of functional FICTCs, and HIV screenings at PHCs/SCs post-intervention. Further it has led to the initiation of HIV screening by village nurses immediately following their training. The intervention has progressively reduced the gap between the registration of pregnant women for ANC and those screened for HIV.

The integration approach also has led to task shifting for HIV screening from MSACS staff to NRHM staff. Several studies in other developing countries indicate that such task shifting –including utilizing primary level staff, such as nurses, for counseling and HIV testing at rural health facilities leads to a higher level of acceptance from the community [19] and is an effective strategy for HIV treatment and care [20].

In addition, the intervention has shownd that the integration of health programs results in substantially lower project costs as the major cost items, like infrastructure, supplies and human resources, are already covered within the program. Coordination and collaboration across different authorities is the essence of this program; however unlike other stand-alone programs, such an integrated program uses existing mechanisms and systems to achieve the stated objectives.

As shown in this study, it is possible to bring the majority of ANC attendees under the HIV prevention umbrella by integrating vertical government programs (HIV with MCH programs). The main advantage is the early detection of HIV infection among pregnant women, providing them an opportunity to access a range of available HIV care, support and treatment services and eventually preventing the transmission of infection to the next generation. An additional benefit is that all ANC attendees will receive pre-test HIV counseling, thereby empowering women with the knowledge needed to live an HIV-free life.

The many gains of such an integrated approach can also be garnered from the fact that based on the same design; the diagnosis and management of sexually transmitted infections were scaled up in Satara district the following year. These results corroborate earlier findings on the integration of such programs [21-24] and the cost-effectiveness of universal HIV screening of pregnant women [25-27].

This study is important given the lack of evidence demonstrating the integration of the PPTCT program with the broader health mission in India and elsewhere. Thestudy results should be considered in light of certain limitations. First, the use of indirect indicators to measure the benefits of the integrated approach from district authorities and several individuals limits generalizability of the results to a larger population level. Second, it was difficult to measure and attribute the independent effects of multiple intervention strategies used by the program for HIV screening. Third, other events promoting HIV testing that occurred concurrently during the intervention period may have influenced the outcome. Fourth, this design lacks a control group; therefore we could only demonstrate change and not attribution. Despite these limitations, the results show that a low incremental cost can have several advantages for HIV prevention effort with integration.

## Conclusions

Integrating HIV screening with the broader NRHM is a promising opportunity to scale up the PPTCT program. However, advocacy, sensitization, capacity building and the judicious utilization of available resources are key to widening the reach of the PPTCT program in India and elsewhere.

## Competing interests

The authors declare that they have no competing interest.

## Authors’ contributions

RD led the overall strategic planning, costing and implementation. SVB contributed to the customization of the technical design, assisted in the planning, implementation, analyzed data and wrote the manuscript. IG conceptualized and designed the cost analyses and revised the manuscript. NS and VR provided guidance to the analytical approach and revised the manuscript. SR assisted with the analysis and revised the manuscript.DD provided programmatic insight and edited the manuscript. SSG designed the concept for increasing the uptake of HIV screening. All authors have read and approved this final submitted manuscript.
